# Nontypeable *Haemophilus influenzae* biofilms: role in chronic airway infections

**DOI:** 10.3389/fcimb.2012.00097

**Published:** 2012-07-25

**Authors:** W. Edward Swords

**Affiliations:** Department of Microbiology and Immunology, Wake Forest School of Medicine, Winston-SalemNC, USA

**Keywords:** biofilms, chronic obstructive pulmonary disease, *Haemophilus influenzae*, otitis media, rhinosinusitis

## Abstract

Like many pathogens inhabiting mucosal surfaces, nontypeable *Haemophilus influenzae* (NT*Hi*) forms multicellular biofilm communities both *in vitro* and in various infection models. In the past 15 years much has been learned about determinants of biofilm formation by this organism and potential roles in bacterial virulence, especially in the context of chronic and recurrent infections. However, this concept has not been without some degree of controversy, and in the past some have expressed doubts about the relevance of NT*Hi* biofilms to disease. In this review, I will summarize the present information on the composition and potential role(s) of NT*Hi* biofilms in different clinical contexts, as well as highlight potential areas for future work.

## Introduction

Biofilms are generically defined as multicellular microbial communities, often encased within a matrix material, which promote persistence within an environment (Costerton et al., 1995, 1987). It is now recognized that many different microbes exist in biofilms, and that the majority of persistent infections involve biofilms (Donlan, 2001, 2002; Bakaletz, 2007; Hall-Stoodley and Stoodley, 2009). Notably, biofilm communities are inherently resistant to antimicrobials and immune effectors due to multiple factors that can include lack of penetration of the biofilm matrix as well as reduced or halted metabolism of the bacteria residing within a biofilm (Fux et al., 2005; Hall-Stoodley and Stoodley, 2009).

*Haemophilus influenzae* is a commensal and opportunistic pathogen that is highly adapted to the human airway that is its primary environment (Erwin and Smith, 2007). The majority of *H. influenzae* strains in carriage and localized disease are the nontypeable *H. influenzae* (NT*Hi*) strains that lack polysaccharide capsules, and are thus completely unaffected by the protective immune response generated by the *Hib* conjugate vaccine (Murphy and Apicella, 1987; Agrawal and Murphy, 2011). NT*Hi* strains can persist within the airways for long periods of time during which carriage is mostly asymptomatic in healthy people (Mukundan et al., 2007). However, in circumstances where host mucosal clearance mechanisms are compromised or impaired, NT*Hi* can cause an array of opportunistic infections that include rhinosinusitis, bronchitis, pharyngitis, and Otitis Media (OM) (Erwin and Smith, 2007).

Biofilm formation by NT*Hi* has been the subject of a considerable amount of work and at least some degree of controversy. While there has been considerable progress on defining determinants of biofilm formation *in vitro* and, to some degree, the consequences of biofilms for persistence and pathogenicity *in vivo*, some have also expressed skepticism about whether NT*Hi* biofilms have significance to disease (Dohar, 2007; Moxon et al., 2008). In this review I will highlight the current state of knowledge regarding determinants of biofilm formation by NT*Hi*, as well as evidence regarding the relevance of biofilms to persistence of this organism *in vivo*.

### Clinical evidence

Over the past 15 years, there have been a large number of studies that implicate biofilms in persistent infections caused by *H. influenzae* (Table 1). OM is an extremely common pediatric ailment that occurs in large part due to dysfunction of the Eustachian tube, resulting in impaired mucosal drainage of the middle-ear chamber and resulting infection with bacterial opportunists, including NT*Hi*, that normally reside in the nasopharynx. While NT*Hi* and other bacteria may be frequently isolated from patients with OM, middle-ear effusions from a large proportion of these patients do not yield culturable bacteria. However, in a series of important early studies, Post and colleagues showed that NT*Hi* and other bacteria could be detected in these samples by PCR-based methods (Post et al., 1995, 1996a,b; Aul et al., 1998; Bakaletz et al., 1998; Liederman et al., 1998). Similarly, middle-ear effusion samples were shown to contain bacterial components (Dingman et al., 1998) and transcripts, indicating bacterial metabolic activity (Rayner et al., 1998). Subsequent analysis of tympanostomy tubes and tissues from patients with chronic/recurrent OM and tissues from experimentally infected chinchillas clearly demonstrated surface-attached bacterial biofilm communities of NT*Hi* and other OM-related opportunists (Post, 2001; Hall-Stoodley et al., 2006; Hoa et al., 2009, 2010). Similar results have been obtained from examination of adenoids from children with chronic or recurrent OM (Hoa et al., 2009; Nistico et al., 2011), as well as in nasal tissues from patients with rhinosinusitis (Foreman et al., 2009, 2011; Oncel et al., 2010). In the context of airway infections associated with chronic obstructive pulmonary disease (COPD), Murphy and Kirkham showed that NT*Hi* peroxiredoxin-glutaredoxin (*pgdX*) was expressed *in vivo* as evidenced by presence of antibody in patient sera (Murphy et al., 2005). Notably, levels of *PgdX* were shown to be increased in NT*Hi* biofilms as compared to planktonic cultures, and mutants defective in *pgdX* in four different NT*Hi* strain backgrounds were shown to have significant impairment in biofilm formation using a static assay (Murphy et al., 2005). While much work remains to be done on this subject, it is clear that the available evidence strongly suggests the presence of biofilms within the lungs of patients with COPD. Moreover, the increase in glutaredoxin/peroxiredoxin levels may indicate that NT*Hi* bacteria within biofilm are under oxidative stress, which is consistent with recent findings from our laboratory related to the stress-response factor Dps (Pang et al., 2012).

**Table 1:** **Clinical presentations of *Haemophilus influenzae* with a biofilm component**.

**Presentation**	**Finding**	**Reference**
Otitis media	Bacteria and bacterial components present in culture-negative effusion fluids	Post et al., 1995, 1996a,b; Dingman et al., 1998
	Bacterial RNA found in culture-negative effusion fluids	Rayner et al., 1998
	*H. influenzae* biofilms in middle-ear chamber of experimentally infected chinchillas	Post, 2001
	*H. influenzae* surface-attached communities in patient tissues	Hall-Stoodley et al., 2006; Hoa et al., 2009, 2010
Chronic bronchitis	Long-term persistence as evidenced by recurrent sputum cultures	Sethi et al., 2002; Murphy et al., 2004
	Expression of peroxiredoxin levels similar to those observed in biofilm	Murphy et al., 2005
Rhinosinusitis	*H. influenzae* surface-attached communities in patient tissues	Foreman et al., 2009, 2011; Oncel et al., 2010

### NETs and NT*Hi* biofilms

One of the criticisms that has been raised regarding NT*Hi* biofilms is the potential for killing by neutrophil extracellular traps (NETs) (Moxon et al., 2008). We thus performed experiments to address the potential role of NETs in biofilm formed during experimental OM infections (Hong et al., 2009). Using immunofluorescent staining and confocal laser scanning microscopy, we showed that NT*Hi* bacteria are found within multicellular biofilm clusters within NET structures. Importantly, these NET/exudate masses were not correlated with clearance of NT*Hi*, as bacterial counts within chinchilla middle-ear cavities exhibiting macroscopically visible biofilms were significantly higher than those found within ears with no visible biofilm (Hong et al., 2009). NT*Hi* bacteria and bacterial components were shown to initiate NET formation, and the bacteria were shown to be highly resistant to killing by NET and additional incoming neutrophils (Juneau et al., 2011). Thus, the observation of surface-attached NT*Hi* bacteria *in vivo* is not likely to represent bacteria that are in the process of being cleared within a NET structure.

### Antibiotics and *H. influenzae* biofilms

As has been observed for many bacterial species, *H. influenzae* bacteria within a biofilm are inherently resistant to antibiotics. For example, Slinger and colleagues demonstrated that *H. influenzae* biofilms are resistant/tolerant to a wide variety of clinically relevant antibiotics (Slinger et al., 2006). Starner and colleagues showed that NT*Hi* isolates from patients with cystic fibrosis formed biofilm communities on immortalized Calu-3 cells, which were highly resistant to treatment with gentamicin (Starner et al., 2006). Notably, this group later demonstrated that lower concentrations of a variety of antibiotics significantly stimulated biofilm formation by NT*Hi* (Starner et al., 2008). Thus, antibiotics may impact both NT*Hi* biofilm formation and resistance of bacteria to biofilms.

### Nt*Hi* surface components and biofilm

Murphy and colleagues examined biofilm formation by a number of NT*Hi* isolates from patients with (COPD) using a static assay. Of note, the strain set examined by this group was one for which extensive data concerning persistence of individual strains within the patient airway, as well as clinical manifestations of disease, were available. While many strains in the set formed significant biofilm, there was no discernable correlation between length of persistence or severity of disease observed (Murphy and Kirkham, 2002). Later work from two different groups showed that sialylation of the bacterial surface promoted biofilm formation by NT*Hi in vitro* in static as well as continuous-flow biofilm systems (Greiner et al., 2004; Swords et al., 2004) and persistence in animal models of OM (Swords et al., 2004; Jurcisek et al., 2005). Sialylation in biofilm was shown to involve a specific subset of sialyltransferases (Jurcisek et al., 2005) and inactivation of a TRAP-family sialic acid transporter was shown to ablate sialylation and NT*Hi* survival within biofilms *in vitro* (Allen et al., 2005). Addition of phosphorylcholine to the bacterial surface was also shown to be increased in biofilm (West-Barnette et al., 2006), and to promote biofilm formation in continuous-flow systems (Hong et al., 2007b) as well as persistence *in vivo* (Hong et al., 2007a). This modification was also shown to significantly modulate host inflammatory responses in both *in vitro* cell culture systems (West-Barnette et al., 2006) and in the chinchilla OM infection model (Hong et al., 2007a).

NT*Hi* also express type IV pili on their surfaces that mediate twitching motility and transformation-related uptake of DNA (Bakaletz et al., 2005). As in other species, the pilus was shown to be essential for biofilm formation by NT*Hi* (Jurcisek and Bakaletz, 2007; Carruthers et al., 2012), and antibodies directed against pili are protective even against established NT*Hi* biofilms (Novotny et al., 2009). Like many bacteria, NT*Hi* bacteria also produce extracellular DNA that is important to biofilm formation (Jurcisek and Bakaletz, 2007; Izano et al., 2009). The nuclear DNA-associated protein DNABIII has an important structural role in stabilizing the extracellular DNA in the NT*Hi* biofilm matrix, and recent work demonstrates that antibody against this factor can not only collapse biofilm structure but also mediate protection and clearance of established NT*Hi* biofilms (Goodman et al., 2011). This work offers a particularly novel target for vaccination against biofilms formed by NT*Hi* and, possibly, other mucosal pathogens. A summary of surface modifications/components associated with NT*Hi* biofilms is provided in Table 2.

**Table 2:** **Summary of current knowledge about determinants of *H. influenzae* biofilm formation**.

**Topic**	**Current knowledge**	**Reference(s)**
Surface components	Subset of LOS glycoforms	Swords et al., 2004; Greiner et al., 2004; Hong et al., 2007a,b
	Sialylated non-LOS carbohydrate	Jurcisek et al., 2005
	Extracellular DNA	Jurcisek and Bakaletz, 2007; Izano et al., 2009
	Pili	Jurcisek and Bakaletz, 2007; Jurcisek et al., 2007
Quorum signaling	Promotes biofilm formation and persistence *in vivo*	Armbruster et al., 2009
	Interspecies quorum signaling	Armbruster et al., 2010; Armbruster and Swords, 2010
	Uptake determinant defined	Armbruster et al., 2011

### Quorum signaling in nontypeable *H. influenzae* biofilms

Like many other pathogens, *H. influenzae* genomic sequences contain a homolog of the *luxS* genetic determinant of production of the so-called interspecies autoinducer-2 quorum signal (Harrison et al., 2005). Autoinducer-2 is a generic term for a family of derivatives of dihydroxypentanedione (DPD), which is produced by many species as a byproduct of homocysteine metabolism. For many species, the AI-2 signal can mediate density-dependent quorum signal events that coordinate communal responses in bacterial populations (Schauder et al., 2001; Waters and Bassler, 2005). The first studies on quorum signaling by *H. influenzae* were performed by Daines and colleagues, who showed that mutation of *luxS* impacted the severity of OM disease but did not abolish the formation of biofilms (Daines et al., 2005). Later, our group expanded on this work to show that while *luxS* mutants did form biofilms, there were significant decreases in surface phosphorylcholine levels, and related decreases in biofilm thickness and density, and persistence in the chinchilla OM infection model (Armbruster et al., 2009). In this work, both genetic and physiologic complementation were shown to restore biofilm formation by *luxS* mutants (Armbruster et al., 2009); this was an extremely important control experiment, as these mutations can have metabolic implications (Vendeville et al., 2005; Rickard et al., 2006). Recently, our group has also demonstrated that, as in other species, RbsB can mediate uptake of DPD for NT*Hi* strain 86-028NP (Armbruster et al., 2011); mutants lacking *rbsB* were also shown to have biofilm defects and decreased persistence *in vivo* comparable to *luxS* mutants (Armbruster et al., 2011).

Our recent work also shows that the AI-2 quorum signals from NT*Hi* promote biofilm formation and persistence of *Moraxella catarrhalis*, an opportunistic pathogen that inhabits the same mucosal environment within the airways (Armbruster et al., 2010). NT*Hi* and *M. catarrhalis* formed polymicrobial biofilms that significantly enhanced antibiotic resistance and bacterial persistence within the chinchilla infection model (Armbruster et al., 2010). Taken together, these data suggest that effective inhibition of quorum signaling could be a potential means to treat OM (Armbruster and Swords, 2010). A summary of current knowledge regarding NT*Hi* quorum signaling and its role in biofilm maturation is provided in Table 2.

### Summary and remaining questions

**Figure 1:**
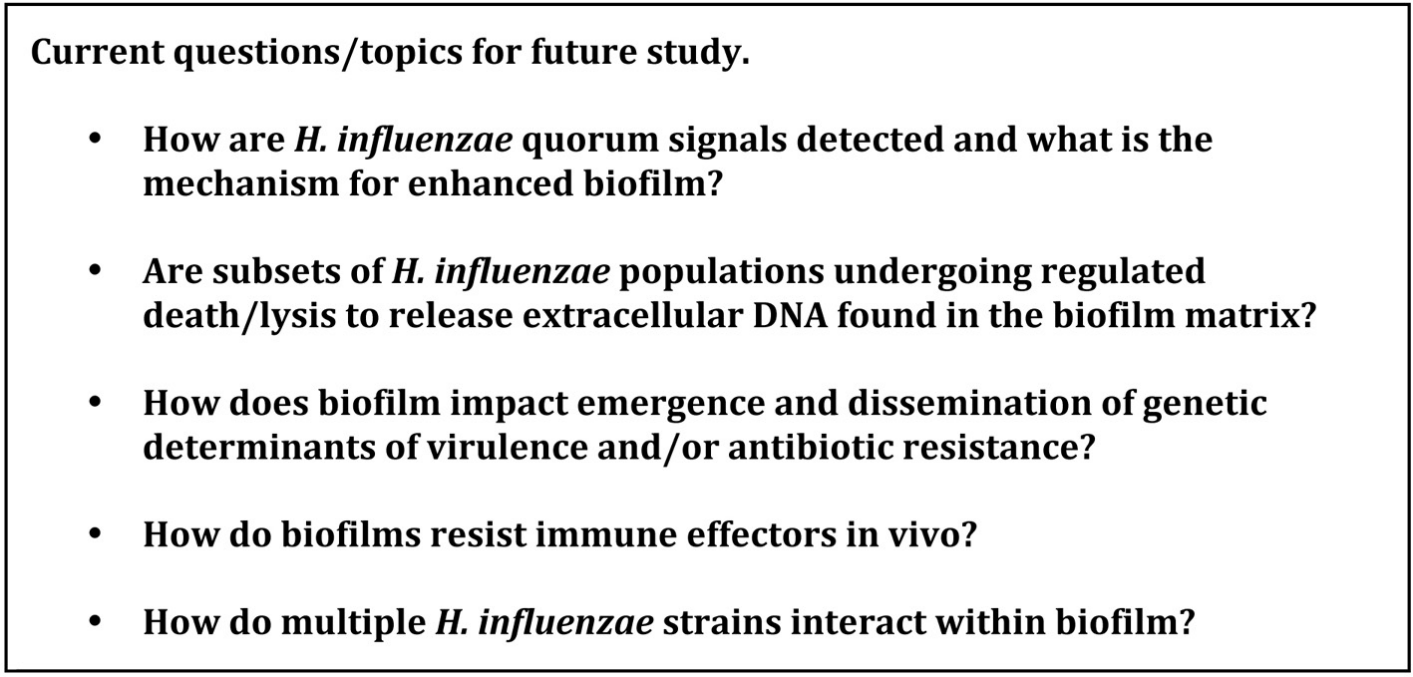
**Current questions/topics for future study**.

Clearly, we have learned much in the past 15 years about the determinants of biofilm formation by *H. influenzae* as well as the role(s) of these biofilms during airway infections. A listing of major remaining questions is provided in Figure 1.

Certainly, there remains a significant amount to be learned about the process of biofilm formation by *H. influenzae*, and persistence of *H. influenzae* bacteria therein. However, a deeper understanding of this mode of bacterial growth seems likely to offer opportunities for new treatment modalities aimed at chronic and recurrent infections.

### Conflict of interest statement

The author declares that the research was conducted in the absence of any commercial or financial relationships that could be construed as a potential conflict of interest.
